# Evaluation of nasal symptoms induced by platelet activating factor, after nasal challenge in both healthy and allergic rhinitis subjects pretreated with rupatadine, levocetirizine or placebo in a cross-over study design

**DOI:** 10.1186/1710-1492-9-43

**Published:** 2013-11-01

**Authors:** Rosa Muñoz-Cano, Antonio Valero, Ignacio Izquierdo, Jaume Sánchez-López, Alejandro Doménech, Joan Bartra, Joaquim Mullol, Cesar Picado

**Affiliations:** 1 Allergy Unit, Pneumology and Allergy Department, Hospital Clínic, Barcelona, Catalonia, Spain; 2 Centro de Investigaciones Biomédicas en Red de Enfermedades Respiratorias (CIBERES), Barcelona, Catalonia, Spain; 3 Global Allergy and Asthma European Network (GA2LEN), Barcelona, Catalonia, Spain; 4 Institut d’Investigacions Biomèdiques August Pi i Sunyer, Barcelona, Catalonia, Spain; 5 Clinical Development Department, J Uriach y Compañia, S.A, Barcelona, Catalonia, Spain; 6 Unitat de Rinologia i Clinica de l’Olfacte, Servei d’Otorinolaringologia, Hospital Clínic, Barcelona, Catalonia, Spain; 7 J Uriach y Compañía, S.A., Poligono Industrial Riera de Caldes, Avinguda, Camí Reial, 51-57, 08184, Palau-Solità i Plegamans, Catalonia, Spain

**Keywords:** Allergic rhinitis, Antihistamines, Healthy volunteers, Levocetirizine, Platelet activating factor, Rupatadine

## Abstract

**Background:**

Platelet-activating factor (PAF) is produced by most inflammatory cells and it is involved in inflammatory and allergic reactions. We aimed to assess the anti-PAF effects of rupatadine and levocetirizine in the upper airways.

**Findings:**

Healthy volunteers (HV, N = 10) and seasonal allergic rhinitis (SAR, N = 10) asymptomatic patients were treated out of the pollen season with either rupatadine 20 mg, levocetirizine 10 mg, or placebo once a day during 5 days prior to the PAF nasal challenge. Total 4-nasal symptom score (T4SS) and nasal patency (Vol_2-5_, by acoustic rhinometry) were assessed from 0 to 240 minutes after a repeated PAF challenge. In SAR patients but not in HV, both rupatadine and levocetirizine showed a trend to decrease PAF-induced T4SS from 60 to 120 minutes. Rupatadine but not levocetirizine caused a significant reduction (p < 0.05) of T4SS area under the curve compared to placebo. Rupatadine and levocetirizine caused no significant changes on nasal patency compared to placebo.

**Conclusions:**

These results suggest that both rupatadine and levocetirizine showed a tendency decrease toward nasal symptoms, but only rupatadine significally reduces the overall nasal symptoms (AUC) induced by PAF in SAR patients.

## Findings

Anti-PAF effects of rupatadine (20 mg) and levocetirizine (10mg) in healthy volunteers (HV, N=10) and seasonal allergic rhinitis (SAR, N=10) asymptomatic patients were evaluated after PAF nasal challenge.

Nasal symptom score (T4SS) and nasal patency (Vol_2-5_) were assessed from 0 to 240 minutes after a repeated PAF challenge.

● In SAR patients but not in HV, both rupatadine and levocetirizine showed a trend to decrease PAF-induced T4SS from 60 to 120 min in comparison with placebo.

● Rupatadine but not levocetirizine caused a significant reduction (p<0.05) of the overall nasal symptoms (AUC from 30 to 240 min) induced by PAF in SAR patients.

● No significant changes of nasal patency were observed in comparison with placebo.

### Background

In addition to other inflammatory mediators, histamine and PAF have a relevant participation in allergic inflammation. Therefore, blocking both PAF and histamine effects might represent a greater clinical efficacy than just blocking one [1]. Rupatadine has a dual anti inflammatory effect by blocking both histamine H_1_ and PAF receptors [2]. As a result, nasal provocation with PAF would allow the evaluation of both clinical and inflammatory nasal response after pharmacological treatment with anti-PAF drugs. In previous investigations, PAF nasal provocation models have shown contradictory results in assessing the patophysiological mechanisms of allergic rhinitis due in part to their lack of sensitivity and specificity [3-5]. Recently, we have investigated the role of PAF in nasal symptoms by means of a human model of PAF nasal challenge in both healthy volunteers (HV) and seasonal allergic rhinitis (SAR) asymptomatic patients out of the pollen season [6]. Long-lasting effects on nasal symptoms were shown after PAF nasal challenge, mainly in nasal obstruction, in both HV and SAR patients.

The aim of the present study was to assess and compare the ability to block the nasal clinical response induced by PAF, both in HV and SAR patients pretreated with rupatadine or levocetirizine.

### Methods

#### Study population

Twenty subjects, 10 HV and 10 SAR asymptomatic patients, were recruited. All SAR patients had positive prick test and serum specific IgE to grass or tree pollen, along with compatible personal history of SAR. All subjects were not allowed to use any medication (antihistamines and/or corticosteroids) for 4 weeks prior to and during the study. All SAR patients were asymptomatic at the inclusion and the study was performed out of pollen season. The study was approved by the Ethics Committee of our institution and informed consent was obtained from all subjects participating before the study.

An Independent Ethics Committees from Hospital Clínic i Provincial (Barcelona, Spain) reviewed and approved the protocol and amendments, the subject’s informed consent document, and related subject information and recruitment materials before the start of the study.

#### Study design

A proof of concept randomized, double-blind crossover study was designed. All subjects were randomized to receive either rupatadine 20 mg, levocetirizine 10 mg, or placebo once daily during 5 days prior the PAF nasal challenge. A washout period of at least 15 days was set between treatment periods.

**Figure 1:**
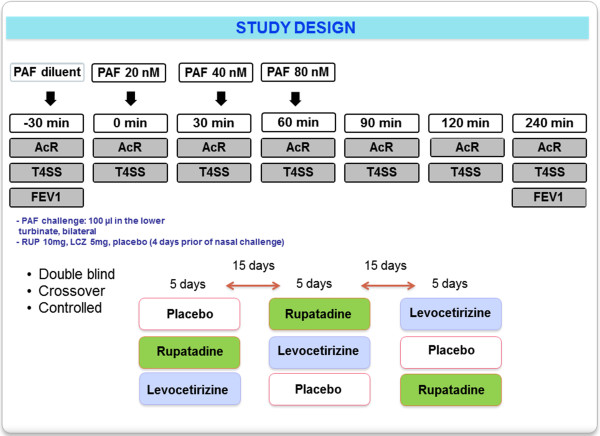
**Squeme and design of study.** PAF: platelel-activating factor; AcR: acoustic rhinometry; T4SS: total 4-symptoms score (evaluated by Likert and visual analogic scales) FEV1: Forced expiratory volume in 1 second.

First, the subjects were challenged with the drug solvent (4% ethanol) thirty minutes before PAF administration (0 minutes) to rule out unspecific nasal reactivity. Three consecutive increasing doses of PAF (20, 40, and 80 nmols, Sigma Aldrich, Madrid, Spain) were instilled with a pipette (100 μl) into each nostril at 0, 30, and 60 minutes (Figure 1). PAF doses were selected based on a pilot test and previously reported studies [3-5].

#### Clinical outcomes

Total 4-symptom score (T4SS) including rhinorrhea, nasal congestion, nasal itching, and sneezing were scored by the subjects by means of a visual analogue scale (VAS, 0–100 mm) and a Likert scale (0 to 3 for each symptom and from 0 to 12 for T4SS). Changes from baseline (time 0) in T4SS were measured at 30, 60, 90, 120, and 240 minutes of the first PAF nasal challenge. Additionally, the Area Under the Curve (AUC) of the T4SS from 30 to 240 minutes was calculated and both treatments were compared versus placebo.

Nasal patency was also evaluated by acoustic rhinometry (AcR) (SER 2000; RhinoMetrics, Lynge, Denmark) in both the right and left nasal cavities between the 2^nd^and 5^th^cm (Vol_2–5_)[7].

#### Statistics

VAS and Lickert scale results were expressed as mean ± SD. Nasal volume changes were expressed as the percentage change from the nasal volume obtained with diluent challenge. Data were compared using ANOVA test and Student’s t-test for paired and unpaired data. The values of p < 0.05 were considered statistically significant.

### Results

**Figure 2:**
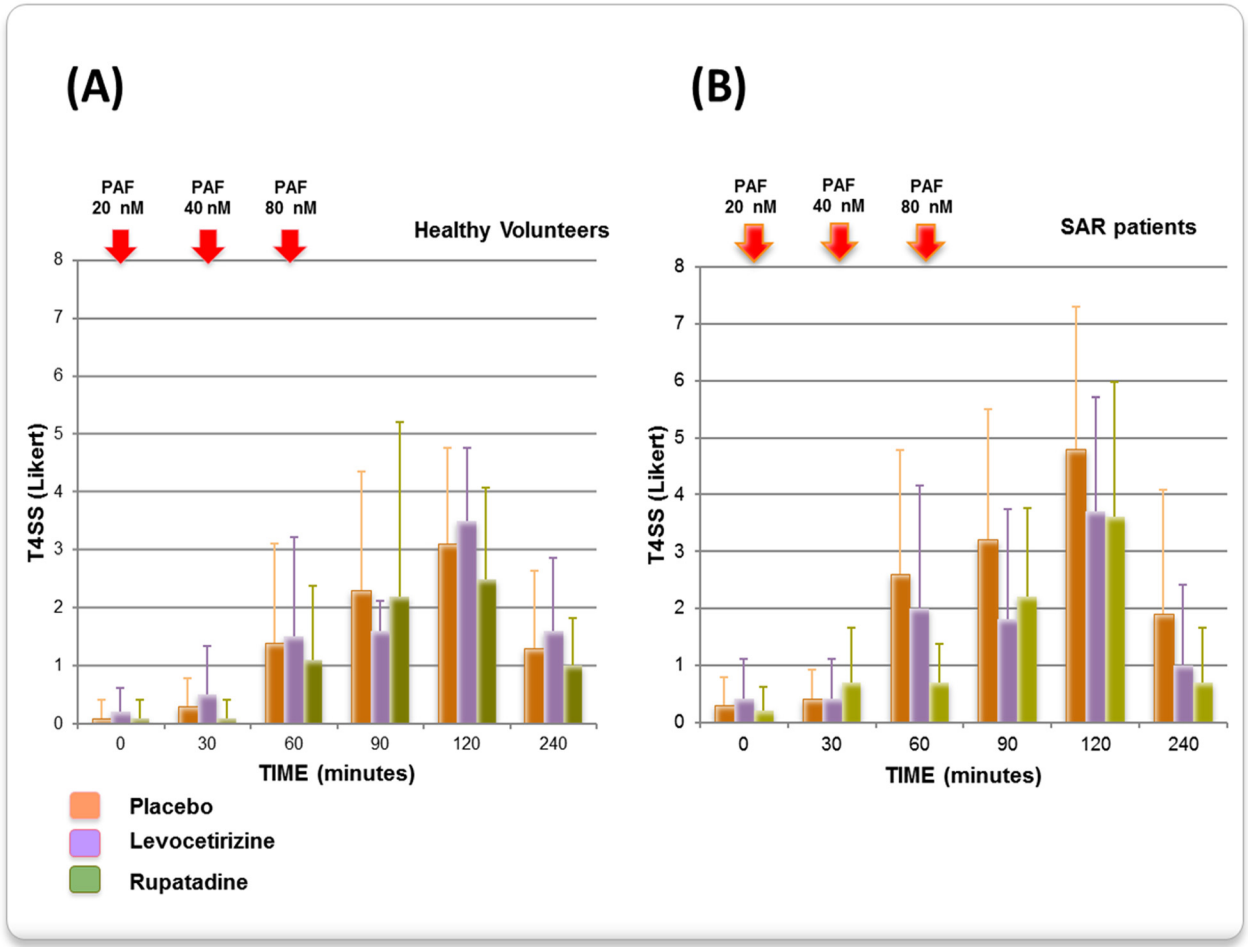
Time-evolution on total nasal symptom score (T4SS) after repeated platelet-activating factor (PAF) nasal challengue, evalueted by Likert scale: (A) in healthy subjects, and (B) in seasonal allergic rhinitis (SAR) patients.

**Figure 3:**
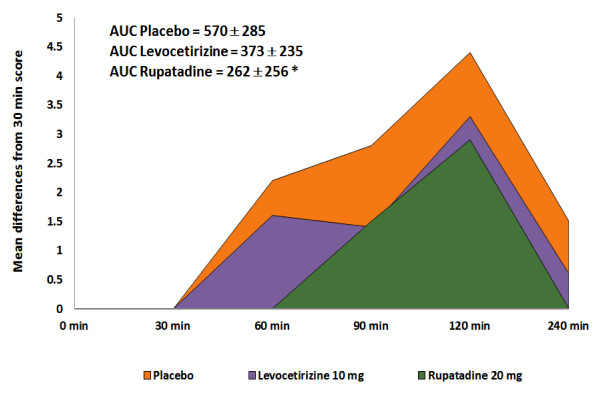
Area under of curve of nasal symptoms (AUC) of T4SS time-course adjusted by baseline values at 30 min.

No significant decreases of T4SS after PAF nasal challenge, using either the Likert or VAS scales, were observed at any time-point for the active treatments in comparison with placebo, and in both HV and SAR patients. Nevertheless, rupatadine caused a 73% decrease compared to placebo of the T4SS (Likert scale) at 60 minutes after PAF in SAR patients. In comparison, levocetirizine produced only a 23% inhibitory effect at 60 minutes (Figure 2). This trend shown by rupatadine was confirmed by the T4SS AUC. Rupatadine showed a statistically significant 54% reduction compared to placebo (AUC score: 262.5 versus 570, p < 0.05) (Figure 3). The T4SS AUC of levocetirizine versus placebo was non-statistically significant.

A progressive decrease in AcR was observed, nevertheless there were no differences between treatment groups in the AUC time-course of nasal patency (Vol_2-5_) after PAF challenge in both HV and SAR patients.

Subjects participating in the study did not report any adverse event related to PAF challenge or the treatment with rupatadine, levocetirizine or placebo.

### Discussion

In this proof of concept study, an increase of nasal symptoms after nasal challenge with PAF was observed. Rupatadine and levocetirizine showed a trend towards decreasing nasal symptoms mainly at 60 minutes after PAF challenge. Nevertheless, statistical differences were not detected probably due to the small sample size and also by the high variability of measurements observed. Statistical significant differences were achieved after evaluating overall time-course of symptoms (AUC). Rupatadine but not levocetirizine significantly reduced AUC values in SAR patients but not in healthy volunteers. In contrast, the nasal patency assessed by AcR did not show any significant changes between the three groups, even after the comparison of AUC assessments.

The precise mechanism of the rupatadine anti-PAF effect is not completely known, although it is thought to be linked to its capacity of blocking the PAF receptors [2,8-10]. PAF stimulates nasal mast cells [11] and has the capability to attract and activate neutrophils and eosinophils [12-14]. Thus, using this human nasal challenge model we could demonstrate the activity of drugs with the ability of blocking PAF receptors and reduce their proinflammatory properties. Antihistamines have the ability to stabilize the inactive form of the receptor of histamine (inverse agonists), partially inhibiting paracrine/autocrine effects of histamine. In this way, the second generation antihistamines, reduce the intensity and duration of allergic symptoms [15]. Levocetirizine and rupatadine are capable of displaying antinflammatory effects through its activities on the histamine receptors. Perhaps such inhibition is not sufficient after a repeated nasal PAF challengue and we could postulate that rupatadine can provide additional antiinflamamatory effects by means of its dual capacity of blocking histamine and PAF receptors in comparison with levocetirizine [16,17]. However, further studies with a higher sample size at different rupatadine dose levels should be carried out to confirm these findings.

In conclusion rupatadine and levocetirizine showed a trend towards the reduction of nasal symptoms after nasal challenge with PAF compared with placebo. A statistically significant inhibitory effect was found only with rupatadine when the AUC time-course of total nasal symptoms was assessed in SAR patients. To our knowlenge, this is the first evidence of PAF inhibitory effects on human nasal airways using an antihistamine drug with dual H_1_ and PAF receptor antagonist activities. Nevertheless, further studies should be performed with a major number of patients with a high and unique dose of PAF challenge in order to reduce the variability of data.

## Abbreviations

AcR: Acoustic rhinometry; AUC: Area under the curve of nasal symptoms; HV: Healthy volunteers; PAF: Platelet-activating factor; SAR: Seasonal allergic rhinitis; T4SS: Total 4-nasal symptom score; VAS: Visual analogue scale.

## Competing interests

Antonio Valero is medical advisor for Stallergenes, FAES, Chiesi, Novartis, and Esteve. He is involved in educational activities sponsored by FAES, Novartis, and Grupo Uriach. Joaquim Mullol has been or is member of national and international scientific advisory boards (consulting), received fees lectures, and grants for research projects from Boheringer-Ingelheim, Esteve, FAES, Hartington Pharmaceuticals, Johnson & Johnson, MEDA, MSD, Novartis, Pierre-Fabre, Shering Plough, UCB, Grupo Uriach, and GSK. Alejandro Domenech and Iñaki Izquierdo are employed of J. Uriach y Compañia, S.A. Rosa Muñoz-Cano, Jaume Sánchez-López, and Joan Bartra have not declared any conflict of interest. Cesar Picado has been or is member of national and international scientific advisory boards (consulting), received fees lectures, and grants for research projects from Esteve, FAES, MSD, Novartis, Chiesi, Grupo Uriach, and GSK.

## Authors’ contributions

RM, AV, JS, and JB were responsible of the conception and design of study, recruitment subjects and interpretation of data. II and AD were responsible for the experimental design, monitoring process, statistics and preparation of drafting the manuscript. JM and CP were responsable of acquisition of funding, general supervision, and supervised, read, and approved the final manuscript. All authors read and approved the final manuscript.

## Authors’ information

Joaquim Mullol and Cesar Picado with senior responsabilities.
